# Editorial: Novel materials, devices and solutions for brain-inspired sensing and computing

**DOI:** 10.3389/fnins.2022.1099962

**Published:** 2022-11-29

**Authors:** Christian Monzio Compagnoni, Shimeng Yu, Weisheng Zhao, Daniele Ielmini

**Affiliations:** ^1^Dipartimento di Elettronica, Informazione e Bioingegneria, Politecnico di Milano, Milan, Italy; ^2^School of Electrical and Computer Engineering, Georgia Institute of Technology, Atlanta, GA, United States; ^3^School of Integrated Circuit Science and Engineering, Fert Beijing Research Institute, Beihang University, Beijing, China

**Keywords:** brain-inspired computing, brain-inspired sensing, neural networks, device and materials engineering, device and materials physics, neuromorphic circuits, solid-state devices

The brain is capable of solving complicated tasks, such as one-shot learning, abstraction and sensory motor control, with an extremely low power consumption of few tens of Watts. This is possible by (i) the large connectivity among the computing elements within the brain, where every neuron is, on average, connected to about 10,000 other neurons, (ii) the spike-based communication/processing protocol, where energy is consumed only when/where it is required, (iii) the co-localization of memory and computing functionalities, and (iv) the high specialization of each individual building block, such as somas, synapses and dendrites. Brain-inspired circuits aim at mimicking the efficient structure and operation of the brain to perform a variety of complex tasks including, learning, adaptation and smart interaction with the environment. In the neuromorphic hardware, realistic artificial counterparts for the biological actors in the human brain structure and operation are needed. Conceiving and developing those counterparts represent an ambitious goal, requiring the investigation and the exploitation of novel materials, devices, and solutions.

This Research Topic presents some of the innovations that are currently being explored to develop artificial components capable of mimicking the biological building blocks of the brain involved in sensing and computing. These innovations can serve as a starting point for the design of complex neuromorphic systems targeting artificial intelligence and high-performance machine learning.

The contributions to this Research Topic cover the following areas:

- advanced materials or material properties for neuromorphic hardware for sensing and computing
- novel solid-state devices for neuromorphic circuits for sensing and computing
- new physical effects in materials and solid-state devices exploitable for neuromorphic hardware for sensing and computing
- innovative operating schemes for mainstream and emerging solid-state devices allowing to reproduce principles and functionalities needed in neuromorphic hardware for sensing and computing

This Research Topic includes four articles, whose content is briefly summarized in the following paragraphs.

In the paper by Cheng R. et al., entitled “*Toward learning in neuromorphic circuits based on quantum phase slip junctions*,” the use of superconducting Quantum Phase Slip Junctions (QPSJs), an electromagnetic dual to Josephson Junctions (JJs), in neuromorphic circuits is discussed. QPSJ-only as well as hybrid QPSJ + JJ circuits are proposed for a variety of neuromorphic applications, including artificial synapses and neurons. Learning circuits based on the proposed artificial neural elements are designed and simulated, exploiting a spike timing dependent plasticity rule to achieve learning. The results presented in the paper will contribute to the development of high-speed and low-power spiking neural networks that are capable of both supervised and unsupervised learning.

In the paper by Yap et al., entitled “*Voltage-time transformation model for threshold switching spiking neuron based on nucleation theory*,” a voltage-time transformation model (V-t Model) to predict and simulate the spiking behavior of threshold-switching selector-based neurons (TS neurons) is presented. The behavior of TS neurons based on different TS devices, including ovonic threshold switching (OTS), insulator-metal transition, and silver-based selectors, is simulated using the proposed V-t model. The results suggest that the OTS neuron is the most promising and potentially achieves the highest spike frequency of GHz and the lowest operating voltage and area overhead. The results presented in the paper provide an engineering pathway toward the future development of TS neurons for neuromorphic computing applications.

In the paper by Lee et al., entitled “*Linear frequency modulation of NbO*_2_*-based nanoscale oscillator with Li-based electrochemical random access memory for compact coupled oscillatory neural network*,” the effect of device parameters of Insulator-Metal Transition (IMT) device-based oscillators with non-volatile analog memory on coupled oscillators network for classification of clustered data is investigated. The results show that the NbO_2_-based IMT oscillator with non-volatile Li-based electrochemical random access memory (Li-ECRAM) has the potential for high network performance thanks to linear conductance modulation characteristics. A coupled oscillator network for spoken vowel classification exploiting an NbO_2_-based IMT device and Li-ECRAM is then studied and shown to be capable of achieving a high classification accuracy (85%), higher than that of a ring oscillator-based system. The results presented in the paper demonstrate that NbO_2_-based oscillators with Li-ECRAM are promising candidates for area-scalable and energy-efficient networks with high performance.

In the paper by Cheng L. et al., entitled “*A bioinspired configurable cochlea based on memristors*,” a novel strategy for building artificial cochleas is proposed, which has a strong potential for configurable, highly-parallel, and highly-efficient auditory systems for neuromorphic robots. A memristor with the TiN/HfO_x_/TaO_x_/TiN structure is exploited to build a filter circuit to implement the cochlea function. By programming the memristor into different resistance values, the artificial cochlea can output signals with specific frequencies and gains. To demonstrate an example of its practical application, the artificial cochlea system is combined with a convolutional neural network to identify 10 classes of audio signals in the Free Spoken Digit Dataset. The results show that the recognition accuracy reaches 92% when the cochlea has 64 memristor-based filtering channels. The results of the paper have a significant potential for robotic sensing applications.

## Author contributions

All authors listed have made a substantial, direct, and intellectual contribution to the work and approved it for publication.

## Conflict of interest

The authors declare that the research was conducted in the absence of any commercial or financial relationships that could be construed as a potential conflict of interest.

## Publisher's note

All claims expressed in this article are solely those of the authors and do not necessarily represent those of their affiliated organizations, or those of the publisher, the editors and the reviewers. Any product that may be evaluated in this article, or claim that may be made by its manufacturer, is not guaranteed or endorsed by the publisher.

